# Seasonal fluctuation of in vitro fertilization encounters in the United States

**DOI:** 10.1007/s10815-023-02777-0

**Published:** 2023-03-21

**Authors:** Alexandra M. McGough, Kaitlin A. Doody, Olivia B. Foy, Chelsey A. Harris, Rachel S. Mandelbaum, Koji Matsuo, Richard J. Paulson

**Affiliations:** 1 Division of Gynecologic Oncology, Department of Obstetrics and Gynecology, University of Southern California, 2020 Zonal Avenue, IRD520, Los Angeles, CA 90033 USA; 2 Keck School of Medicine, University of Southern California, Los Angeles, CA USA; 3 Division of Reproductive Endocrinology and Infertility, Department of Obstetrics and Gynecology, University of Southern California, Los Angeles, CA USA; 4 Norris Comprehensive Cancer Center, University of Southern California, Los Angeles, CA USA

**Keywords:** In vitro fertilization, Seasonal fluctuation, Patient factor, Hospital factor

## Abstract

**Purpose:**

To examine patient and hospital characteristics related to seasonal fluctuation in in vitro fertilization (IVF).

**Methods:**

This retrospective cohort study examined 33,077 oocyte retrievals identified in the National Ambulatory Surgery Sample. Exposure assignment was monthly IVF encounters: low-volume months (<25%ile), mid-volume months (≥25/<75%ile), and high-volume months (>75%ile). Main outcomes were patient and hospital characteristics related to the exposure groups, assessed with a multinomial regression model.

**Results:**

The median IVF encounters were 977 per month, ranging from 657 to 1074 (absolute-difference 417). January, July, and December were the lowest-quartile volume months, ranging from 657 to 710 encounters per month (low-volume months). May, August, and November were the top-quartile volume months, ranging from 1049 to 1074 encounters per month (high-volume months). In a multivariable analysis, patients undergoing IVF in the low-volume months were younger and less likely to have infertility or comorbidities. Patients undergoing IVF in the high-volume months were more likely to have lower household income and receive IVF at urban teaching facilities. Northeastern residents were less likely to have IVF in the low-volume months but more likely to have IVF in the high-volume months. Sensitivity analyses showed that the lowest-to-highest variability in monthly IVF encounters was higher in Northeast region compared to other regions (320 vs 50–128); infertility patients compared to those without (317 vs 190); privately insured patients compared to self-pay (227 vs 156); and older patients compared to younger (234 vs 192).

**Conclusion:**

This study suggests substantial seasonal fluctuation in IVF oocyte retrieval in the USA based on patient and hospital factors.

**Supplementary Information:**

The online version contains supplementary material available at 10.1007/s10815-023-02777-0.

## Introduction

Seasonal patterns in natural conceptions in humans have been widely documented across a multitude of different populations across the world [1]. It is speculated that this may be due to temperature variation or environmental factors but is also likely influenced by cultural and behavioral factors. In the USA, particularly among southern states, a pattern involving a peak of births in September and a trough in April–May has been reported [2].

In vitro fertilization (IVF) provides a tightly controlled environment that may reduce the seasonal effects seen in unassisted pregnancies [3]. The question of whether there may be seasonal fluctuations in IVF success has been frequently debated with varying and contradictory results [4–10]. Many previous studies have found no seasonal effects on fertilization, pregnancy, and implantation rates [4–6], while others have found variations in implantation and clinical pregnancy [7–9] as well as live birth depending on outdoor temperature [10, 11]. The studies that observed seasonal variation in IVF success found these differences to be most pronounced between winter and summer [7, 10].

A very important question when considering the existing data on seasonal fluctuation in IVF success is who chooses to undergo IVF when, as this may affect patterns in clinical pregnancy and live birth rates. Seasonal fluctuation in patient and hospital factors has been reported in other diseases which affect health outcomes [12, 13]. Seasonal fluctuation in IVF patient encounters has not been well studied previously; however, it is an important lens with which to view existing epidemiologic data regarding the seasonality of fertility and IVF success. The objective of the current study was therefore to examine seasonal fluctuation in IVF utilization in the USA.

## Methods

### Data source

This retrospective cohort study queried the Healthcare Cost and Utilization Project’s Nationwide Ambulatory Surgery Sample (NASS) [14]. NASS is the largest all-payer database for ambulatory surgery centers in the USA. The NASS program collects information for ambulatory surgeries performed in hospital-owned facilities. In 2019, nearly 9 million encounters, estimating 11.8 million encounters for national-level statistics, were collected across 2958 facilities. This data capture schema of NASS represents approximately 68% of ambulatory surgeries in US hospital-owned facilities. The University of Southern California Institutional Review Board deemed this study exempt due to the use of publicly available, deidentified data.

### Study population

The study population included encounters for oocyte retrievals from January 2016 to December 2019. The identification of oocyte retrieval procedures was based on the American Medical Association’s Current Procedural Terminology code 58970. This code does not include the procedure for embryo transfer to the uterus. Patients with unknown age and missing information for month of encounter were excluded. Encounters for 2018 were also not used due to a change in the program’s in-scope criteria in this year.

### Intervention

The exposure assignment of the study population was based on the number of IVF encounters in each month from January to December. First, monthly IVF encounters were generated in three calendar years examined (Supplemental Figure S1). In each month, average IVF encounters were then computed. These twelve calendar months were then quantified based on the monthly IVF encounters to ≤25%ile, 26–50%ile, 51–75%ile, and >75%ile.

Patients who had IVF in the lowest-quartile months were assigned as the low-volume month group. Patients who had IVF in the highest-quartile months were assigned as the high-volume month group. Patients who had IVF in the remaining months were assigned as the mid-volume month group.

### Outcome measures

The main outcome measures were independent patient and hospital characteristics across the exposure assignment. Specifically, characteristics of IVF encounters in low-volume months and high-volume months were compared to mid-volume months.

### Study variables

Among the eligible patients for analysis, patient demographics and hospital parameters were abstracted from the NASS program. Patient demographics included age at IVF encounter (<37 and ≥37 years) dichotomized per the median, primary expected payer (private including HMO, self-pay, and other), median household income for patient’s ZIP code (every quartile), patient location (large central metropolitan, large fringe metropolitan, medium metropolitan, small metropolitan, micropolitan, and not metropolitan or micropolitan counties), female infertility (yes or no), polycystic ovary syndrome (yes or no), and Charlson comorbidity index (0 or ≥1) [15]. The World Health Organization’s International Classification of Disease 10th revision (ICD-10) of N97 and E28.2 were used to identify female infertility and polycystic ovary syndrome, respectively.

Hospital parameters included relative hospital bed capacity (small, medium, and large), hospital location and teaching setting (urban non-teaching or urban teaching), and hospital region (Northeast, Midwest, South, and West). The hospital parameters were determined per the NASS program.

### Analytic approach

Descriptive statistical analysis was used to describe cohort-level characteristics based on the median value with interquartile range or frequency with percentage. Patient and hospital characteristics across the exposure groups were analyzed using a multinomial regression model fitted for multivariable analysis. All the measured study covariates were entered in the final model. The mid-volume month group served as the reference group, and the effect size for the low-volume month group and the high-volume month group were expressed with adjusted-odds ratio (aOR) with a corresponding 95% confidence interval (CI).

Sensitivity analysis included evaluation of the inter-month variability of IVF encounters based on demographic characteristics. The absolute difference between the highest and lowest monthly IVF encounters was computed. The analysis was performed for age (<37 and ≥37 years), primary expected payer (private and self-pay), infertility diagnosis (yes and no), and hospital region (Northeast, Midwest, South, and West). These factors were chosen in a post hoc fashion based on the findings of primary outcome measures.

The weighted values for national estimates provided by the NASS program were utilized for statistical analysis. Statistical interpretation was based on a two-tailed hypothesis, and a *P*<0.05 was considered statistically significant. Cases with missing information were grouped as one category in each variable. IBM SPSS Statistics (version 28.0, Armonk, NY, USA) was used for all analysis. The STROBE reporting guidelines were followed to summarize the performance of the cohort study [16].

## Results

A total of 33,077 IVF encounters were examined for analysis. The cohort-level characteristics are summarized in Supplemental Table S1. The median age was 37 (interquartile range 33–40) years. The majority of patients were residents of large central metropolitan areas (60.7%), had high household income (highest quartile, 59.6%), and had a diagnosis of infertility (67.9%). Private insurance (48.6%) was the most common type of primary expected payer, followed by self-pay (32.0%). Patients in this cohort were most frequently located in Northeastern US (37.8%). More than 90% of IVF encounters occurred in the setting of large, urban teaching facilities.

**Fig. 1:**
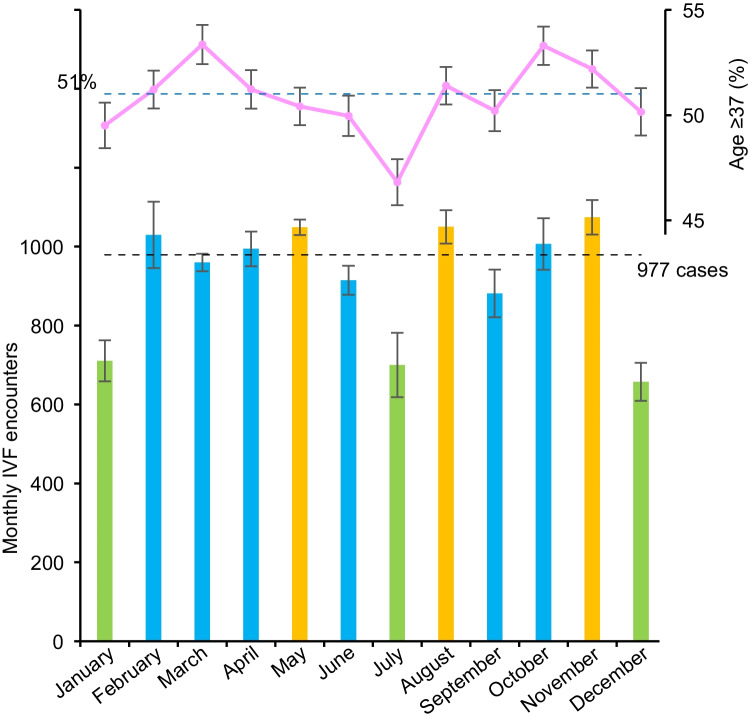
Monthly distribution of IVF encounters. Monthly number of IVF encounters (bottom) and proportion of patients aged ≥37 years (top) from January to December are shown. Orange color indicates the top-quartile encounter months, and light green color indicates the lowest-quartile encounter months. Black horizontal dashed line indicates the median of monthly IVF encounters a month (977). Blue horizontal dashed line indicates the cohort-level proportion of patients with ≥37 years (51%). Error bars indicate standard error. Abbreviation: IVF, in vitro fertilization

The monthly distribution of IVF encounters is shown in Fig. 1. The median number of IVF encounters per month was 977, ranging from 657 encounters in December (lowest) to 1074 encounters in November (highest). The absolute difference from the lowest to highest months was 417 encounters.

The lowest-quartile volume group consisted of the months January, July, and December, ranging from 657 to 710 encounters a month (low-volume months). In contrast, May, August, and November comprised the top-quartile volume group, ranging from 1049 to 1074 encounters a month (high-volume months). The remaining second-third quartile comprised the mid-volume months, ranging from 881 to 1030. This assignment was consistent in each calendar year (Supplemental Figure S1).

Volume-specific patient characteristics across the low-, mid-, and high-volume months are shown in Table 1. The number of IVF encounters was 6202 in the low-volume months, 17,355 in the mid-volume months, and 9519 in the high-volume months.

**Table 1:** Patient demographics per exposure (*N*=33,077)

Characteristic	Low-volume months	Mid-volume months	High-volume months
Definition	January, July, December	**	May, August, November
No.	*n*=6202	*n*=17,355	*n*=9519
Age (year)			
<37	51.2	48.4	48.7
≥37	48.8	51.6	51.3
Primary expected payer			
Private	49.0	48.7	48.2
Self	33.2	31.6	31.9
Other	14.1	14.1	14.8
Unknown	3.8	5.6	5.1
Household income			
QT1 (lowest)	8.6	8.5	8.9
QT2	11.6	11.0	11.5
QT3	19.8	19.7	18.0
QT4 (highest)	58.7	59.6	60.4
Unknown	1.3	1.2	1.3
Patient location			
Large central metropolitan	58.9	60.7	61.9
Large fringe metropolitan	20.8	20.1	20.3
Medium metropolitan	9.6	9.2	8.7
Small metropolitan	5.3	4.7	4.2
Micropolitan	2.6	3.0	2.4
Not metropolitan/micropolitan	12.1	21.6	1.6
Unknown	0.7	0.6	0.8
Female infertility			
No	34.3	31.2	32.2
Yes	65.7	68.8	67.8
Charlson comorbidity index			
0	97.1	96.8	97.2
≥1	2.9	3.2	2.8
Polycystic ovary syndrome			
No	97.7	98.2	98.1
Yes	2.3	1.8	1.9
Hospital region			
Northeast	31.0	38.0	42.0
Midwest	30.6	27.5	23.9
South	10.8	10.5	9.6
West	27.7	24.1	24.5
Hospital teaching			
Urban non-teaching	0.5	0.5	0.3
Urban teaching	99.5	99.5	99.7
Hospital bed capacity			
Small	0.8	0.9	0.8
Mid	4.8	3.7	3.8
Large	94.4	95.5	95.5

Percentage per column is shown. **Remaining months: February, March, April, June, September, and October. *QT* quartile

In a multivariable analysis (Table 2), when compared to the patients undergoing IVF in the mid-volume months, patients undergoing IVF in the low-volume months were less likely to be ≥ 37 years of age (aOR 0.92, 95% CI 0.87–0.98). This was particularly prominent in IVF encounters in July (Fig. 1). Patients undergoing IVF in the low-volume months were also less likely to have a diagnosis of infertility (aOR 0.91, 95% CI 0.85–0.97) or comorbidity (aOR 0.83, 95% CI 0.69–0.98), and they were less likely to reside in micropolitan areas (aOR 0.82, 95% CI 0.68–0.99). IVF encounters in the low-volume months more likely occurred in medium bed capacity facilities (aOR 1.51, 95% CI 1.05–2.17). Northeastern US residents were less likely to undergo IVF in the low-volume months (aOR 0.73, 95% CI 0.67–0.79).

**Table 2:** Multivariable analysis

	Low-volume vs mid-volume		High-volume vs mid-volume	
Characteristic	aOR (95% CI)	*P*-value	aOR (95% CI)	*P*-value
Age (year)				
<37	1.00 (reference)		1.00 (reference)	
≥37	0.92 (0.87–0.98)	0.008	0.96 (0.92–1.01)	0.150
Primary payer				
Private	1.00 (reference)		1.00 (reference)	
Self	1.04 (0.97–1.11)	0.277	1.00 (0.94–1.06)	0.920
Other	1.10 (1.00–1.20)	0.051	1.01 (0.93–1.09)	0.786
Unknown	0.85 (0.73–0.99)	0.049	0.81 (0.71–0.91)	<0.001
Household income				
QT1 (lowest)	0.99 (0.88–1.11)	0.831	1.16 (1.05–1.27)	0.004
QT2	1.03 (0.93–1.15)	0.527	1.14 (1.04–1.24)	0.004
QT3	0.96 (0.89–1.04)	0.327	0.98 (0.91–1.05)	0.615
QT4 (highest)	1.00 (reference)		1.00 (reference)	
Unknown	0.96 (0.64–1.43)	0.832	0.96 (0.67–1.37)	0.817
Patient location				
Large central metropolitan	1.00 (reference)		1.00 (reference)	
Large fringe metropolitan	1.02 (0.94–1.10)	0.714	1.07 (1.00–1.14)	0.064
Medium metropolitan	0.98 (0.88–1.09)	0.695	1.01 (0.92–1.12)	0.773
Small metropolitan	1.04 (0.90–1.20)	0.604	0.99 (0.87–1.13)	0.879
Micropolitan	0.82 (0.68–0.99)	0.035	0.84 (0.71–0.99)	0.040
Not metropolitan/micropolitan	1.13 (0.90–1.41)	0.287	1.02 (0.83–1.25)	0.884
Unknown	1.20 (0.71–2.04)	0.497	1.43 (0.90–2.27)	0.128
Infertility				
No	1.00 (reference)		1.00 (reference)	
Yes	0.91 (0.85–0.97)	0.003	0.98 (0.92–1.03)	0.384
Charlson comorbidity				
0	1.00 (reference)		1.00 (reference)	
≥1	0.83 (0.69–0.98)	0.029	0.91 (0.78–1.06)	0.224
Polycystic ovary syndrome				
No	1.00 (reference)		1.00 (reference)	
Yes	1.12 (0.92–1.38)	0.265	1.14 (0.94–1.38)	0.179
Region				
Northeast	0.73 (0.67–0.79)	<0.001	1.12 (1.05–1.20)	0.001
Midwest	0.97 (0.88–1.06)	0.501	0.83 (0.76–0.90)	<0.001
South	0.89 (0.79–1.01)	0.062	0.87 (0.78–0.96)	0.009
West	1.00 (reference)		1.00 (reference)	
Hosp teaching				
Urban non-teaching	1.00 (reference)		1.00 (reference)	
Urban teaching	0.91 (0.60–1.38)	0.659	1.63 (1.08–2.46)	0.020
Hosp bed capacity				
Small	1.00 (reference)		1.00 (reference)	
Mid	1.51 (1.05–2.17)	0.026	1.18 (0.86–1.61)	0.315
Large	1.19 (0.84–1.68)	0.329	1.03 (0.76–1.39)	0.856

A multinomial regression model for multivariable analysis. Mid-volume months (2nd/3rd quartiles) served as the referent group. *aOR* adjusted-odds ratio, *CI* confidence interval, *QT* quartile

Patients in the high-volume months were more likely to have lower household income (aOR for the lowest-quartile income 1.16, 95% CI 1.05–1.27; and aOR for the second lowest-quartile income 1.14, 95% CI 1.04–1.24) and reside in micropolitan areas (aOR 0.84, 95% CI 0.71–0.99) compared to the patients in the mid-volume months. IVF encounters in the high-volume months more likely occurred in urban teaching facilities (aOR 1.63, 95% CI 1.08–2.46). Northeastern US residents were more likely to undergo IVF in high-volume months (aOR 1.12, 95% CI 1.05–1.20) while those in Midwest/South regions were less likely (aOR 0.83, 95% CI 0.76–0.90, and aOR 0.87, 95% CI 0.78–0.96, respectively) (Table 2).

**Fig. 2:**
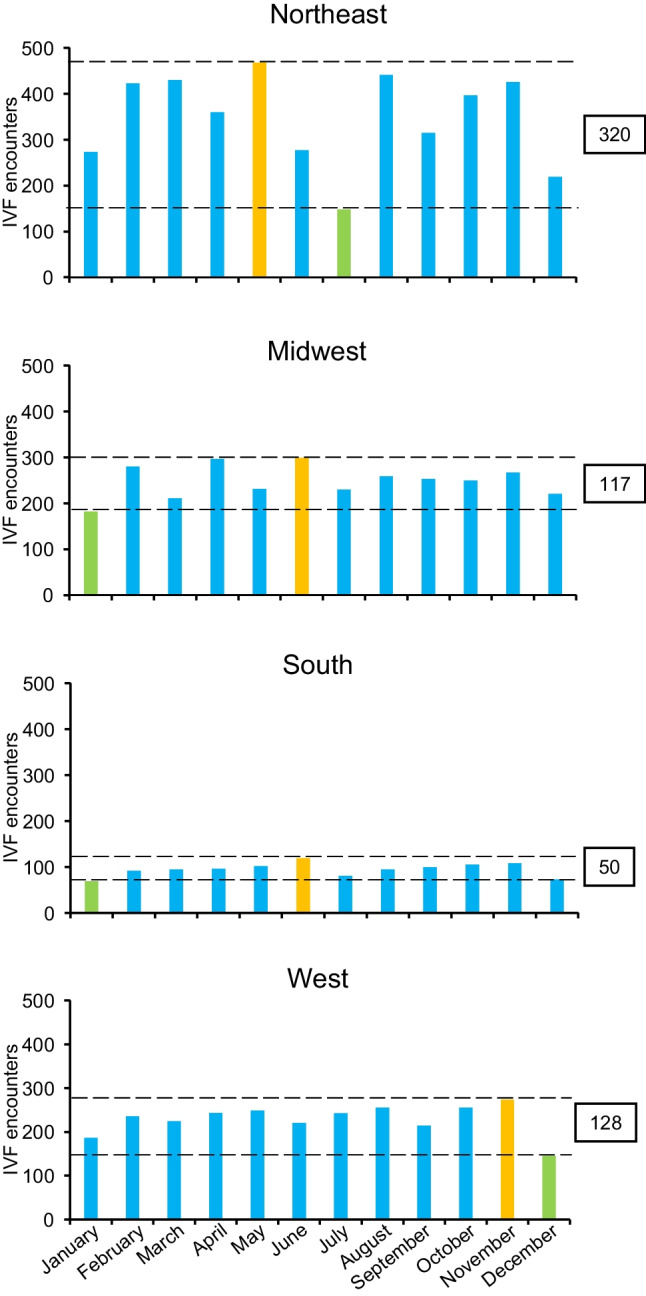
Region-specific monthly IVF encounters. Orange colors indicate the highest encounter month, and light green color indicates the lowest encounter month. The seasonal fluctuation from the highest and lowest encounters are shown in the boxes between the two horizontal dashed lines

Monthly distributions of IVF encounters per US region are shown in Fig. 2. The Northeast region had the highest median IVF encounters a month followed by Midwest, West, and South (378, 251, 239, and 95 encounters a month, respectively). The variability of monthly IVF encounters between the lowest and highest months was 320 in the Northeast, which was a few times higher than that of other regions (128 in West, 117 in Midwest, and 50 in South, respectively; net-difference 270).

Monthly distributions of IVF encounters per patient age are shown in Supplemental Figure S2. The median number of monthly IVF encounters was 464 for patients aged <37 years and 510 for those with aged ≥37. The highest-lowest difference in monthly IVF encounters were higher in the age ≥37 group compared to the age <37 group (234 *versus* 192; net-difference 42).

Monthly distributions of IVF encounters per primary expected payer are shown in Supplemental Figure S3. The median number of monthly IVF encounters was 475 for patients with private insurance and 313 for those with self-pay. The highest-lowest variability in monthly IVF encounters was higher in the privately insured group compared to the self-pay group (227 *versus* 156; net-difference 71).

Monthly distributions of IVF encounters per infertility status are shown in Supplemental Figure S4. The median number of monthly IVF encounters was 668 for patients with the diagnosis of infertility and 292 for those without. The highest-lowest variability in monthly IVF encounters were higher in the infertility group compared to the non-infertility group (317 *versus* 190; net-difference 127).

## Discussion

### Principal findings

This study demonstrates substantial variability in IVF encounters for oocyte retrieval throughout the year, which is associated with patient and hospital factors. This seasonal fluctuation was particularly noticeable in winter months (December and January) and early summer (July) during which monthly IVF encounters were 27–33% fewer than the cohort-level median.

### Insights for results

#### Patient factors

Specific patient and lifestyle factors may impact IVF success and conceivably may cause seasonal fluctuations in outcomes. This may be influenced by improved diet, exercise, and vitamin D exposure during the summer compounded by increased air pollution and smog during the winter [7, 10, 17–20]. Patterns in patient choice may also impact seasonal differences in IVF. This may be influenced by the financial accessibility of fertility treatment at different time points in the year, meeting annual insurance deductibles, or holiday and work/vacation schedules. In the West, Midwest, and Northeast, IVF utilization was highest in early summer, while in the West, IVF utilization was highest in November. Similarly, the lowest volume months for the West, Midwest, and South were in the winter months, but in the Northeast, this effect occurred in July.

In the USA, IVF is very costly and only 15 states currently mandate infertility insurance coverage, which are concentrated in the Northeast [21–25]. This may explain higher utilization of IVF in the Northeast, as more patients are likely to use IVF if they reside in a state where infertility insurance coverage is mandated [22–25]. This contrasts with Europe, for example, where IVF is more affordable and no significant seasonal fluctuations have been observed [4–6, 8].

Even with insurance coverage, many patients may be required to attempt a certain number of intrauterine insemination cycles prior to starting IVF. Patients who used insurance for IVF instead of self-paying had almost 1.5 times greater variability between low- and high-volume months (Supplemental Figure S3). With insurance policies generally beginning in January, this delay may explain why there is a trough in IVF use later in the winter and a rise in the summer after patients have completed the requirements to start IVF [26, 27]. IVF use among insured patients peaks in November, perhaps representing couples’ desire to undergo treatment when they have already met their deductible or may be waiting for financial aid (Supplemental Figure S3) [28]. This idea may also explain why patients with lower income are more likely to get IVF during these high-volume months. These patients may rely more heavily on insurance coverage to meet their fertility goals.

Our results demonstrating patients in low-volume months are more likely to be younger, healthier, and less likely to have a diagnosis of infertility could be explained by a shift in the patient demographic at that those times. Patients undergoing graduate or professional training may schedule their IVF encounters for time during school breaks, which align with the low-volume months [29]. This may reflect the egg freezing population, which generally are younger maternal patients with better prognosis. Additionally, various professional schedules may give rise to replicable seasonal use of IVF services such as patients who are teachers, farmers, or dependent on seasonal tourism for work.

#### Hospital factors

We also considered the effect of hospital factors on the variation in IVF use especially during winter months. This may be explained by the existence of short-term fertility laboratory closures for the purposes of cleaning and maintenance coinciding with the holiday-dense winter season in the USA [30]. Laboratory closures necessarily prevent oocyte retrieval and incubation and require a certain period without IVF procedures for the cleaning and maintenance before re-opening. Fertility clinics may also take advantage of the additional influence of insurance coverage changes for their patients during this time [31]. A comparison to fertility clinics outside of the USA would be necessary to fully support this argument.

### Study limitations

There are several limitations in this study. Unmeasured bias is inherent to a retrospective study. The most important confounder that was not captured in the study but may influence the analysis was the shared decision-making process with patient and reproductive endocrinologist in deciding the timing of IVF. IVF cycle (first or repeated) and the indication of IVF for non-female infertility cases (e.g., male infertility, recurrent pregnancy losses, preimplantation genetic testing, and social egg freezing) are other unmeasured confounders that may impact the exposure-outcome association in the study.

Selection bias may exist in this study as the NASS program only captures the IVF encounters performed in the ambulatory surgery setting in hospital-owned facilities. It is speculated that large number of patients undergo IVF in private practices or non-hospital-based settings, which are not captured by this study.

Additionally, outcomes of IVF are not available in the NASS program. Thus, it is unknown if the IVF success rates differ across seasons. We are also unable to confirm the accuracy of the data in the NASS program, as there is no actual medical record review. Last, generalizability in different regions or populations was not assessed in this study.

### Clinical implications

The clinical implications of this study rest in the importance of taking into account the patterns of patients who choose to undergo IVF at specific times when investigating IVF success over time. The observed associations also likely translate to seasonal variability in IVF births.

This study also echoes evidence provided by other proponents of increasing access to IVF services in the USA. Cost-effectiveness analyses have demonstrated that IVF would cause an insignificant increase in insurance policy costs nationwide [26]. States that mandate fertility coverage experience a higher utilization of fertility services [22–25]. European studies have demonstrated that decreased cost of IVF, while not completely sufficient to optimize accessing fertility care, does eliminate some disparities and variations in success rates related to time [24, 32]. These factors together with our demonstration that variations in IVF use may depend on patient access to fertility care further bolsters the urgent need for equal and affordable access to infertility care in the USA.

## Supplementary information

ESM 1 (DOCX 256 kb)

## Declarations

### Disclaimer

The funder had no role in the design and conduct of the study; collection, management, analysis, and interpretation of the data; preparation, review, or approval of the manuscript; and decision to submit the manuscript for publication.

### Transparency

The manuscript’s corresponding author (K.M.) affirms that the manuscript is an honest, accurate, and transparent account of the study being reported; that no important aspects of the study have been omitted; and that any discrepancies from the study as planned (and, if relevant, registered) have been explained. The National Ambulatory Surgery Sample as a part of the Healthcare Cost and Utilization Project (HCUP) by the Agency for Healthcare Research and Quality is the source of the de-identified data used; and the program has not been verified and is not responsible for the statistical validity of the data analysis or the conclusions derived by the study team.
