# Supplementary material for: Seasonal fluctuation of in vitro fertilization encounters in the United States
Source: J Assist Reprod Genet. 2023 Mar 21;40(5):1099–107. doi: 10.1007/s10815-023-02777-0 (PMC10239406; doi:10.1007/s10815-023-02777-0)
Supplement: Supplementary file 1 — (DOCX 256 kb) [file 10815_2023_2777_MOESM1_ESM.docx]

**Supplemental Figure S1. Meta-data for monthly IVF encounters.**


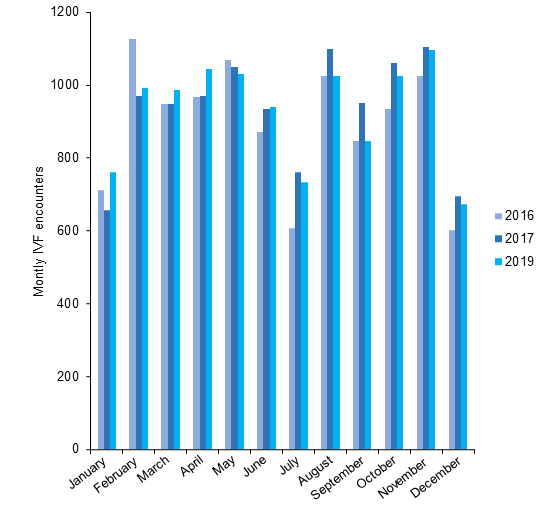


Year-specific data for monthly IVF encounters is shown from January to December in three examined calendar years.

**Supplemental Figure S2. Monthly IVF encounters per patient age.**

**
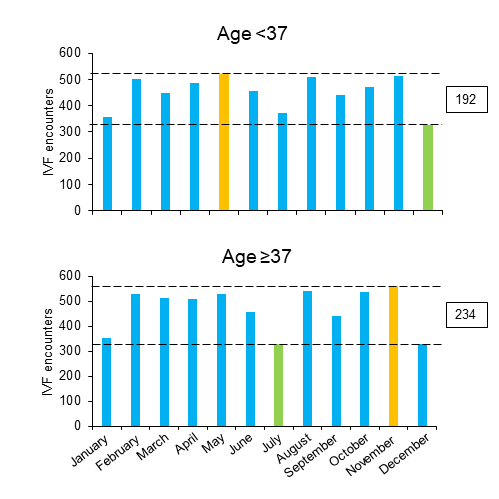
**

Orange bars indicate the highest encounter month, and light green bars indicates the lowest encounter month. The seasonal fluctuation from the highest and lowest encounters are shown in the boxes. Age was grouped according the median value of study cohort.

**
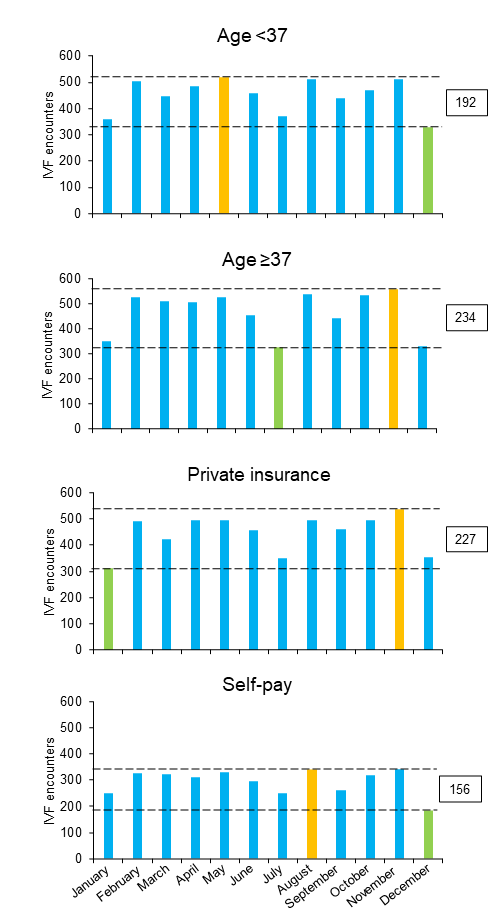
Supplemental Figure S3. Monthly IVF encounters per primary expected payer types.**

Orange bars indicate the highest encounter month, and light green bars indicates the lowest encounter month. The seasonal fluctuation from the highest and lowest encounters are shown in the boxes.


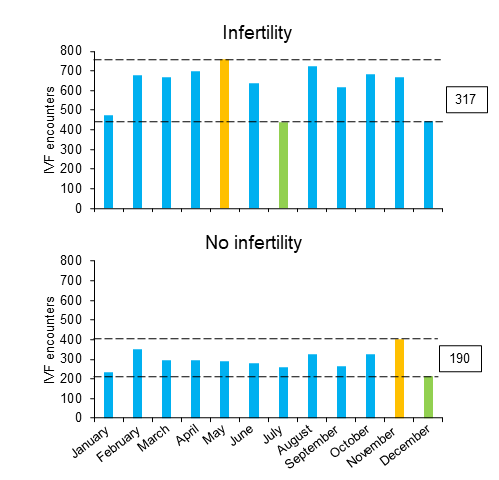
**Supplemental Figure S4. Monthly IVF encounters per infertility diagnosis.**

Orange bars indicate the highest encounter month, and light green bars indicates the lowest encounter month. The seasonal fluctuation from the highest and lowest encounters are shown in the boxes.

**Table 1. Cohort-level characteristics (*N*=33,077).**

| Characteristic | No. (%) |
| --- | --- |
| Age (y) | 37 (33-40) |
| <37 | 16,211 (49.0) |
| ≥37 | 16,865 (51.0) |
| Primary expected payer |  |
| Private | 16,082 (48.6) |
| Self | 10,582 (32.0) |
| Other | 4,726 (14.3) |
| Unknown | 1,686 (5.1) |
| Household income |  |
| QT1 (lowest) | 2,853 (8.6) |
| QT2 | 3,724 (11.3) |
| QT3 | 6,368 (19.3) |
| QT4 (highest) | 19,726 (59.6) |
| Unknown | 406 (1.2) |
| Patient location |  |
| Large central metropolitan | 20,076 (60.7) |
| Large fringe metropolitan | 6,712 (20.3) |
| Medium metropolitan | 3,028 (9.2) |
| Small metropolitan | 1,545 (4.7) |
| Micropolitan | 912 (2.8) |
| Not metropolitan / micropolitan | 566 (1.7) |
| Unknown | 236 (0.7) |
| Infertility |  |
| No | 10,614 (32.1) |
| Yes | 22,462 (67.9) |
| Charlson comorbidity index |  |
| 0 | 32,076 (97.0) |
| ≥1 | 1,000 (3.0) |
| Polycystic ovary syndrome |  |
| No | 32,454 (98.1) |
| Yes | 622 (1.9) |
| Hospital region |  |
| Northeast | 12,518 (37.8) |
| Midwest | 8,930 (27.0) |
| South | 3,400 (10.3) |
| West | 8,228 (24.9) |
| Hospital teaching |  |
| Urban non-teaching | 151 (0.5) |
| Urban teaching | 32,926 (99.5) |
| Hospital bed capacity |  |
| Small | 267 (0.8) |
| Mid | 1,294 (3.9) |
| Large | 31,515 (95.3) |

Number and percentage per column are shown. Total number may not be 33,077 due to weighted value. Abbreviation: QT, quartile.
